# Supplementary material for: Baseline nowcasting methods for handling delays in epidemiological data
Source: Wellcome Open Res. 2026 Feb 14;10:614. Originally published 2025 Nov 5. [Version 2] doi: 10.12688/wellcomeopenres.25027.2 (PMC13005074; doi:10.12688/wellcomeopenres.25027.2)
Supplement: Peer review history

# Reviewer report: Reviewer response for version 2

**DOI:** 10.21956/wellcomeopenres.28633.r147768

**Published:** 2026-03-20

**Authors:** Giorgio Guzzetta

**Affiliations:** 1 Fondazione Bruno Kessler, Trento, Italy

The manuscript has significantly improved in terms of clarity and precision of communication, and I now do not see major issues with the proposed methods and analyses.

 I still think that the claims of the manuscript are overstated, especially in the opening paragraph of the discussion:

- the nowcasting method is not an innovation proposed by the authors (only the R package is).
- the practical use of the baseline nowcasting method for public health would require testing under simulated real-time conditions, whereas the authors admittedly used consolidated data for model training.
- the usability of the package for public health professionals needs to be demonstrated; the claim that "public health co-authors were able to implement the package in their systems" only constitutes anecdotal evidence, not a proper demonstration.

Other minor points below:

- In my opinion, Figure 3 and related results are just a sanity check, not a scientific result, and should be moved to the appendix.
- I still think Figure 4 is overloaded with information (26 panels!) and could benefit from moving non-essential information to the Supplementary Materials. The y label is missing in Figure 4D. 
- A brief explanation of how the overdispersion parameter of the negative binomial observation model is chosen would be helpful.
- P. 7 "We implement a slightly more complex version of this to account for zero reported cases observed so far." -> A short explanation of how this is implemented in the main text is needed, at least in terms of general principles, along with a reference to the supplementary materials.
- Possible typos: 
  - P. 4 "The chain ladder methods [refs], Lawless"
  - P. 17 "[…] random walks [refs], d B-splines"

Is the work clearly and accurately presented and does it cite the current literature?

No

If applicable, is the statistical analysis and its interpretation appropriate?

Not applicable

Are all the source data underlying the results available to ensure full reproducibility?

Yes

Is the study design appropriate and is the work technically sound?

Partly

Are the conclusions drawn adequately supported by the results?

Partly

Are sufficient details of methods and analysis provided to allow replication by others?

No

Reviewer Expertise:

Infectious disease modelling

I confirm that I have read this submission and believe that I have an appropriate level of expertise to confirm that it is of an acceptable scientific standard, however I have significant reservations, as outlined above.

# Reviewer report: Reviewer response for version 2

**DOI:** 10.21956/wellcomeopenres.28633.r150108

**Published:** 2026-03-20

**Authors:** Fatima Dakroub

**Affiliations:** 1 American University of Beirut, Beirut, Lebanon

The study presents a nowcasting approach and accompanying R package to address reporting delays in real-time disease surveillance data. The topic is relevant and valuable for public health practice. The study is generally well-structured, and the methods are clearly written. However, it may benefit from some refinement to improve readability. Below are some minor comments:

 1. Consider replacing the numerical labels for each panel in Figure 2 with letters and referring to these in the legend to improve clarity and make it easier for the reader to follow.

 2. References: The formatting of references is inconsistent, with some citations presented in superscript and others not. Please ensure a uniform citation style is used throughout the manuscript.

 Also consider replacing “three models in Mellor et al.” with “three models reported by Mellor et al.”

 Regarding this sentence, please add the author’s name et al.: “As demonstrated by 5, such approaches can propagate errors and may not fully account for interdependencies between these processes.”

 “GAM implementation in 2,”: you can delete “in”

 3. “Each case record includes a specimen date (when the sample was collected) and a report date (when the test result was ingested into the surveillance system).”

  The term “ingested” may be unclear. Consider replacing it with a more standard phrasing, such as “when the test result was recorded in the surveillance system” to improve clarity for the reader.

 4. “The difference between these dates forms the reporting delay, which typically peaks at 1–2 days with most reports occurring within 7 days.”

 The description of the reporting delay may be unclear, as stating that it “peaks at 1–2 days” while “most reports occur within 7 days” could be misinterpreted as contradictory. Consider rephrasing by first stating that the majority of cases are reported within 7 days, followed by clarifying that the most common delay is 1–2 days.

 Also replace 7 with "seven" (numbers below 10 should be written in text form)

 5. Discussion: 

 •    “Lastly, by providing a standardised R package with comprehensive documentation (refer to reference no. 1)”.

 There is no need to add the link, as it was already referenced in the materials and methods

 •    “Relatedly, co-creation of documentation with public health practitioners” 

 The phrase is not clear; if the authors mean that public health practitioners should be involved in the documentation process alongside researchers, please re-write the phrase more clearly

Is the work clearly and accurately presented and does it cite the current literature?

Yes

If applicable, is the statistical analysis and its interpretation appropriate?

I cannot comment. A qualified statistician is required.

Are all the source data underlying the results available to ensure full reproducibility?

Yes

Is the study design appropriate and is the work technically sound?

Yes

Are the conclusions drawn adequately supported by the results?

Yes

Are sufficient details of methods and analysis provided to allow replication by others?

Yes

Reviewer Expertise:

Public health, active surveillance, infectious diseases

I confirm that I have read this submission and believe that I have an appropriate level of expertise to confirm that it is of an acceptable scientific standard, however I have significant reservations, as outlined above.

## References

[1] : Baseline Nowcasting Tutorials and Function Documentation. *Available at: https://baselinenowcast.epinowcast.org/index.html* .–;

# Reviewer report: Reviewer response for version 1

**DOI:** 10.21956/wellcomeopenres.27585.r142120

**Published:** 2026-01-21

**Authors:** Sandra Flores Alvarado

**Affiliations:** 1 Programa de Bioestadística, Universidad de Chile Escuela de Salud Publica (Ringgold ID: 193077), Independencia, Región Metropolitana, Chile

The article introduces baselinenowcast, an R package designed to provide a standardized, low-complexity baseline for nowcasting epidemiological count data. This tool is highly relevant for public health surveillance, particularly in emergency contexts where rapid implementation and transparency are paramount to inform policy decisions. The package successfully implements a version of the chain-ladder method and provides uncertainty quantification via a negative binomial model. While the software represents a valuable contribution to the field, there are certain methodological and structural aspects that could be further clarified. The following suggestions are intended to help strengthen the manuscript and its practical application for public health users.

 A central point for clarification is the deterministic nature of the initial estimation step. The current approach uses the chain-ladder method to calculate a point estimate of the delay distribution, which is then used as a fixed baseline for the negative binomial model. This assumes that reporting delay proportions remain relatively constant, which may not fully capture the high variability inherent in real-time reporting during an active outbreak. Integrating this stochasticity more directly into the delay estimation process would likely provide a more realistic representation of uncertainty.

 The validation using German COVID-19 data relies on 7-day moving averages rather than raw daily counts. The use of smoothed data removes the daily noise and weekend effects that a nowcasting tool is specifically intended to resolve. Because the data has been pre-processed, the estimated overdispersion parameters and the resulting prediction intervals may reflect the effects of the smoothing rather than the model's inherent ability to handle raw surveillance data. Testing the package with raw daily reports would provide a more robust evaluation of its performance in real-world settings.

 Regarding the comparative analysis, the manuscript uses a revised version of the KIT simple nowcast instead of the official gold standard. Given that several co-authors are also linked to the original KIT model, it is important to maintain a clear distinction between these two projects. The KIT model's implementation issues should ideally be addressed within its own framework, and this paper should focus on an independent validation of baselinenowcast against established, official benchmarks to ensure the objectivity and reproducibility of the results.

 As a software tool article, the manuscript would be more effective if it focused less on the narrative history of the project and more on its technical implementation. Currently, the text lacks specific examples of the package’s architecture, such as function definitions, primary arguments, or code snippets. The claims regarding usability, low computational costs, and minimal expertise would be better supported if the main text provided technical evidence or a clear walkthrough of the functions necessary for a user to implement the tool during an emergency.

 Finally, the visual presentation of Figure 2 could be improved to better guide the reader through the methodology, as the current layout and curve rendering appear unpolished and lack a clear reading order. Additionally, it would be helpful to define how the model handles specific data edge cases, such as instances where a report occurs before the recorded symptom onset date (e.g., asymptomatic cases). Clarifying whether to use the earliest available date or another criterion, along with refining the graphical rendering, would significantly strengthen the manuscript for indexing.

Is the work clearly and accurately presented and does it cite the current literature?

Partly

If applicable, is the statistical analysis and its interpretation appropriate?

Yes

Are all the source data underlying the results available to ensure full reproducibility?

Yes

Is the study design appropriate and is the work technically sound?

Partly

Are the conclusions drawn adequately supported by the results?

Yes

Are sufficient details of methods and analysis provided to allow replication by others?

Partly

Reviewer Expertise:

Biostatistics, epidemiological surveillance, epidemiology.

I confirm that I have read this submission and believe that I have an appropriate level of expertise to confirm that it is of an acceptable scientific standard, however I have significant reservations, as outlined above.

# Response

**Published:** 2026-02-02

**Authors:** Kaitlyn Johnson

**Affiliations:** Infectious Disease Epidemiology, London School of Hygiene and Tropical Medicine Department of Infectious Disease Epidemiology, London, England, UK

Thank you for your helpful feedback. Please find our responses to each point below.    A central point for clarification is the deterministic nature of the initial estimation step. The current approach uses the chain-ladder method to calculate a point estimate of the delay distribution, which is then used as a fixed baseline for the negative binomial model. This assumes that reporting delay proportions remain relatively constant, which may not fully capture the high variability inherent in real-time reporting during an active outbreak. Integrating this stochasticity more directly into the delay estimation process would likely provide a more realistic representation of uncertainty.  

 >Thanks for this suggestion, we agree that there is an important distinction to be made between our approach and alternative approaches based on bootstrapping or errors obtained from combining many different estimated delays. In our methodology, we chose to produce a point nowcast and then estimate a parametric observation model (by default a negative binomial) for each nowcast horizon. However, this is not the only way to do this, for example, some of us at Massachusetts Department of Health developed a similar method which used directly the 95th percentiles on the observed past nowcast errors to compute the current 95% confidence intervals on their nowcast. We have added a point on this in the discussion, stating:   “In this analysis we only considered negative binomial observation models applied to the target dataset, which in this case were always count data. Other methods for uncertainty estimation could have been explored, such as using bootstrapping or computing percentiles directly from past nowcast errors in a non-parametric form, or exploring other parametric observation models. The modular design of the package enables exploration of alternative error models. Other valid error models can be readily explored, given the modular design of our baseline package.”   We also clarified how the uncertainty is estimated in the methods in the uncertainty estimation description.    We agree that there still might be additional high variability inherent in real-time reporting during an outbreak. One of the advantages of our approach is its modularity– so while the user can estimate the negative binomial (or any observation model of their choice) using past retrospective nowcast errors as described above, they don’t have to – they could instead estimate them from another dataset/outbreak or add additional dispersion to the estimates if they believe them to be not fully representative of the expected variability.   

 The validation using German COVID-19 data relies on 7-day moving averages rather than raw daily counts. The use of smoothed data removes the daily noise and weekend effects that a nowcasting tool is specifically intended to resolve. Because the data has been pre-processed, the estimated overdispersion parameters and the resulting prediction intervals may reflect the effects of the smoothing rather than the model's inherent ability to handle raw surveillance data. Testing the package with raw daily reports would provide a more robust evaluation of its performance in real-world settings.  

 >Thanks for bringing this up, we used 7-day sums because this was the epidemiologically relevant surveillance statistic used in Germany and the target for the Nowcast challenge set up and described in Wolffram et al. In our method, we are nowcasting using the individual level data and then summarising that, with the observation model based on the 7-day sum. This does mean that our observations correlated, and in this case, it was shown by Wolffram et al that estimating the observation model on the 7-day sum directly improves performance over estimating for daily data and combining in post-processing.    We clarify this choice in the methods:

 “Because the 7-day rolling sums was the epidemiologically relevant surveillance statistic and the one used by the German Nowcast Hub, we generated nowcasts of the 7-day rolling sum of COVID-19 hospitalisation incidence each day for national-level aggregated data and age-stratified data.”   And add to the Discussion a point about the limitations inherent in the choice of using 7-day rolling sums instead of daily level data:   “Additionally, for the German COVID-19 data we used the 7-day rolling sums because this was what was epidemiologically relevant and used in the Nowcast Hub, however, the use of this target meant that the daily noise and weekday effects were removed, preventing us from evaluating the methods ability to capture these effects. ”   Because nowcasts were produced each day and the input, pre-processed data was on a daily scale, we still were able to evaluate the nowcasts ability to handle weekday effects in reporting – and we did in fact find that allowing for weekday-specific delay distributions improved nowcasts compared to not adjusting for the weekday when computing daily delay distributions (Fig 4B).  

 Regarding the comparative analysis, the manuscript uses a revised version of the KIT simple nowcast instead of the official gold standard. Given that several co-authors are also linked to the original KIT model, it is important to maintain a clear distinction between these two projects. The KIT model's implementation issues should ideally be addressed within its own framework, and this paper should focus on an independent validation of baselinenowcast against established, official benchmarks to ensure the objectivity and reproducibility of the results.  

 >Thanks for flagging this, we agree that there is a need for benchmarks to compare against for nowcasting model development, and the lack thereof is a primary motivation of this analysis. The KIT model was found to be a reasonably well-performing baseline in Wolffram et al, which is why we chose that model to develop into a software package which allows tuning for different settings. In an ideal world, we would have performed our validation against only what was submitted to the Hub in real-time. However, because of the implementation bug, which was a result we found as part of this study, we would not have been able to validate that our implementation was working correctly if we had not fixed the bug and made the comparison. To provide a thorough comparison to official results and ensure our method was working as we expected, we compare to the real-time Hub nowcasts in the SI, and discuss the finding of the bug and our resulting validation, in the main text (Fig 3 main text, and against the original version Fig. S1 & S2).   

 As a software tool article, the manuscript would be more effective if it focused less on the narrative history of the project and more on its technical implementation. Currently, the text lacks specific examples of the package’s architecture, such as function definitions, primary arguments, or code snippets. 

 Thanks for this feedback, we thought that it was better to use this article as an introduction to the project and a validation of its implementation. We think that it is better to demonstrate package code in the package documentation where it can be kept up to date, whereas in a paper which is generally a static document. We agree that more signpost to this is needed and so have added the following signposting to package documentation in the Methods under software implementation:    “ Worked examples applied to common datasets, as well as documentation of package functions and arguments, are provided as part of the package documentation at https://baselinenowcast.epinowcast.org/index.html”   And in the Discussion:

   “Lastly, by providing a standardised R package with comprehensive documentation (see https://baselinenowcast.epinowcast.org/index.html for tutorials and function documentation),”   The claims regarding usability, low computational costs, and minimal expertise would be better supported if the main text provided technical evidence or a clear walkthrough of the functions necessary for a user to implement the tool during an emergency.   We agree that these claims should be justified. We have edited the concluding paragraph of the discussion to reflect the usability, and have removed any reference to lower computational costs (while we do think this is true, we didn’t formally evaluate its speed in this work).    “Our method, developed with public health practitioners, is robust, interpretable, and relatively straightforward, evidenced by our public health co-authors who were able to implement the package in their systems within a few days unsupported. “

 Finally, the visual presentation of Figure 2 could be improved to better guide the reader through the methodology, as the current layout and curve rendering appear unpolished and lack a clear reading order.    Thanks. We have reorganized Figure 2 to make it more logical and have revised the contents to clean up the presentation.      Additionally, it would be helpful to define how the model handles specific data edge cases, such as instances where a report occurs before the recorded symptom onset date (e.g., asymptomatic cases). Clarifying whether to use the earliest available date or another criterion, along with refining the graphical rendering, would significantly strengthen the manuscript for indexing.  

 >Thanks for this feedback. One of the problems is that there are many such edge cases and in the interest of clarifying for readers it is difficult for us to give a complete overview of what can be achieved with the flexibility of the method. We have added a section on dealing with data edge cases in the Supplement (SI Text. Dealing with edge cases in real-world data) and have added the following reference to it in the main text:   “There are a number of ways in which dealing with real-world data can create edge cases and complexities, such as for example when the “reference date” can refer to different primary events, or when reporting delays are registered as being negative.  We discuss these and ways to handle them in this framework in the SI Text. Dealing with edge cases in real world data. “   This section points to package documentation with further examples on how to deal with some of the edge cases like the one mentioned here.

# Reviewer report: Reviewer response for version 1

**DOI:** 10.21956/wellcomeopenres.27585.r140119

**Published:** 2026-01-02

**Authors:** Giorgio Guzzetta

**Affiliations:** 1 Fondazione Bruno Kessler, Trento, Italy

This manuscript presents an R package implementing a previously proposed multiplicative nowcasting algorithm, explores alternative algorithmic specifications, and evaluates performance using COVID-19 hospitalization data from Germany (late 2021–early 2022) and norovirus surveillance data from the UK (late 2023–early 2024). While the topic is timely and the authors’ intent is commendable, the manuscript in its current form does not fully achieve its two stated aims: providing a practical tool for public health practice and serving as a standardized benchmark for methodological research. In addition, assessment of the methods, results, and overall contribution is hindered by substantial issues related to clarity, excessive verbosity, and limited synthesis of insights. At several points, the paper reads more like a technical report, leaving much of the burden of identifying the main messages and implications to the reader.

**Major issues**

**1. Clarity of methods**

 a. The Methods section is excessively verbose and difficult to follow, as it mixes statistical assumptions, implementation heuristics, software design choices, and sensitivity analyses without a clear hierarchy. For example, much of page 7 falls between a package vignette and a sensitivity analysis description but does not fully succeed in either role. Several expressions (e.g., “data ingestion assumptions,” “pipe-friendly interface”) are not clearly defined. For a paper aiming to promote accessibility and practical usability, the exposition is unexpectedly hard to navigate. A clearer separation between the statistical model, default methodological choices, and software implementation details would substantially improve readability.

 b. The authors should more clearly explain the rationale behind points (a) and (b) of Step 1 (Delay estimation, page 5). In particular, there is concern that this procedure may propagate errors over longer delays. This issue could be exacerbated when the reporting delay distribution is not monotonically decreasing, as is often the case in practice (especially for delays between symptom onset or hospitalization and reporting). The nowcasting algorithm appears closely related to that proposed by Lawless (1994), yet this reference is not cited.

 c. It is not clear which data are used to estimate the delay distribution and which are used for nowcasting. For instance, on page 8 the German COVID-19 dataset is described as containing approximately 160 reference dates, with 60 used for delay estimation and 60 for “retrospective nowcasts” (another undefined term). However, Figure 3c appears to report WIS values over the full data domain, creating confusion about the evaluation setup. This applies equally to the norovirus use case and to the age-specific nowcasting applications.

 d. Figure 3e shows time- and age-varying reporting delays, but it is unclear why (or how) this variability is not incorporated into the nowcasting procedure. Additionally, the absolute WIS values shown in Figure 3c provide limited insight into nowcast quality and do not align clearly with the rWIS values discussed in the text.

 e. In both applications, nowcasts are validated using X-day-ahead rolling evaluation datasets. It is unclear why the authors did not instead evaluate predictions against consolidated values from the final version of the reporting dataset.

 Overall, the lack of clarity in methodology and investigation pipeline makes it impossible to determine the robustness of the results.

**2. Figure quality**

 Overall figure quality is substandard. Some plots in Figure 2 appear hand-drawn, while Figures 3, 4, and 5 contain an excessive number of panels, with color schemes and legends that are difficult to map to the corresponding results. For example, in Figure 5, it is hard to assess whether any model provides qualitatively accurate nowcasts and which legend applies to panels on the right.

**3. Information overload**

 The manuscript devotes substantial space to issues of limited scientific relevance, such as the identification of implementation bugs and baseline consistency checks (e.g., Figure 3b,d). Similarly, Figures 4 and 5 and their accompanying text present a large volume of detailed results that are difficult to contextualize, especially given the lack of methodological clarity, and to summarize in its epidemiological meaning. Differences across results of model variants appear minimal, yet they are discussed at considerable length.

**4. Claims and positioning**

 In the introduction, the work positions itself as both a practical tool for public health practice and a standardized benchmark for methodological research. However, as also acknowledged in the Discussion, the paper does not succeed in achieving either objective.

 In summary, the work appears to be a reasonable validation exercise of an existing and previously validated model using COVID-19 data (conditional on the robustness of the methods, which would need to be clarified more clearly). The extension to norovirus data does not appear competitive relative to other existing models. The main added value of the work seems to lie in providing additional flexibility in algorithmic choices, though these are currently described unclearly, and in wrapping the method into an R package, whose usability and practical value remain to be demonstrated.

Is the work clearly and accurately presented and does it cite the current literature?

No

If applicable, is the statistical analysis and its interpretation appropriate?

Not applicable

Are all the source data underlying the results available to ensure full reproducibility?

Yes

Is the study design appropriate and is the work technically sound?

Partly

Are the conclusions drawn adequately supported by the results?

Partly

Are sufficient details of methods and analysis provided to allow replication by others?

No

Reviewer Expertise:

Infectious disease modelling

I confirm that I have read this submission and believe that I have an appropriate level of expertise to state that I do not consider it to be of an acceptable scientific standard, for reasons outlined above.

# Response

**Published:** 2026-02-02

**Authors:** Kaitlyn Johnson

**Affiliations:** Infectious Disease Epidemiology, London School of Hygiene and Tropical Medicine Department of Infectious Disease Epidemiology, London, England, UK

We thank the reviewer for the feedback. We have reorganised the methods section, clarified how training data is used for estimation, and revised the schematic figure to improve its readability. Please find our detailed responses to each review point below.   

 a. The Methods section is excessively verbose and difficult to follow, as it mixes statistical assumptions, implementation heuristics, software design choices, and sensitivity analyses without a clear hierarchy. For example, much of page 7 falls between a package vignette and a sensitivity analysis description but does not fully succeed in either role. Several expressions (e.g., “data ingestion assumptions,” “pipe-friendly interface”) are not clearly defined. For a paper aiming to promote accessibility and practical usability, the exposition is unexpectedly hard to navigate. A clearer separation between the statistical model, default methodological choices, and software implementation details would substantially improve readability.

 > Thanks for this feedback. We agree that the initial methods section interspersed software defaults with methods in a way that wasn’t clear. We revised the methods section to clearly delineate between the high-level description of the model and the default method specifications in the package, options for model specifications, and the details of the software implementation. We also removed the term “data ingestion assumptions”, using instead language around the amount of training data used for delay and uncertainty estimation, and defined the term pipe-friendly interface. We think this should help the reader delineate between the core method used in the package, the defaults, and the features that are tunable, with the description of the sensitivity analyses (validation, comparison of method specifications, and comparison to external methods) going within each separate case study.   

 b. The authors should more clearly explain the rationale behind points (a) and (b) of Step 1 (Delay estimation, page 5). In particular, there is concern that this procedure may propagate errors over longer delays. This issue could be exacerbated when the reporting delay distribution is not monotonically decreasing, as is often the case in practice (especially for delays between symptom onset or hospitalization and reporting). 

 > We thank the reviewer for raising this concern. We have reworded the description of the delay estimation for clarity (see first bullet point of Step 2). We have tested a range of delays both in the paper and in the package development and did not observe error propagation over longer delays.    We agree with the reviewer that there is potential for non-monotonically decreasing CDFs and we highlight this as an area of future work in the Discussion paragraph (second to last paragraph) when we refer to downward revisions. We don’t anticipate that the extension to handle downward revisions will propagate errors. We have also added a section to the SI Text. Dealing with edge cases in real world data which discusses additional real-world data issues, since this is not a focus of the main text.     The nowcasting algorithm appears closely related to that proposed by Lawless (1994), yet this reference is not cited. We appreciate the reviewers suggestion to cite Lawless (1994) and have added it when we describe the nowcasting algorithm upon which the method is based upon. We have also added a citation to Bizzotto et al to reflect that it is an extension of the Lawless method.   

 c. It is not clear which data are used to estimate the delay distribution and which are used for nowcasting. For instance, on page 8 the German COVID-19 dataset is described as containing approximately 160 reference dates, with 60 used for delay estimation and 60 for “retrospective nowcasts” (another undefined term). However, Figure 3c appears to report WIS values over the full data domain, creating confusion about the evaluation setup. This applies equally to the norovirus use case and to the age-specific nowcasting applications.

 >Thanks for pointing out this area lacking clarity. The total reference times used for uncertainty estimation overlaps with those used for delay estimation because the uncertainty is estimated by producing a retrospective nowcast for multiple retrospective nowcast times. At each of those times, the delay is re-estimated, using the same amount of training data specified for delay estimation.   The uncertainty estimation step now reads:    “We generate retrospective point nowcasts (light shaded bars and blue lines in Figure 2, step 3) by repeating steps 1 and 2 for a number of past, retrospective nowcast times, M, using N reference times for delay estimation at each retrospective nowcast time.This requires N + M total reference times, which means that the total reference times used for uncertainty estimation overlaps with those used for delay estimation, as each retrospective nowcast requires re-estimating a delay using N reference times.“   There is a WIS value reported every day in Figure 3C because we are fitting the model and nowcasting for each day i.e time aware cross validation. The value at each time point summarises the score for the nowcast produced on that day. In practice, this means that the delay estimate is “rolling”, as the delay is always based on the latest data available. We have clarified this in the methods section on the German Nowcast Hub validation which now states:    “ We retrained the model and produced nowcasts each day from 22 November 2021 to 29 April 2022, with preliminary data available from 1 July 2021 used for training the earlier models. Because we retrained the model each time we produced a nowcast, the delay estimate is always based on the latest data available as of the time the nowcast is made.”

 d. Figure 3e shows time- and age-varying reporting delays, but it is unclear why (or how) this variability is not incorporated into the nowcasting procedure. Additionally, the absolute WIS values shown in Figure 3c provide limited insight into nowcast quality and do not align clearly with the rWIS values discussed in the text.

 > Thanks for pointing this out. As described above, the delay is estimated at each nowcast date based on the latest data, and we edited the text to clarify this. A larger training volume will lead to less variable (more slowly changing) delay estimates, and a smaller training volume will lead to more variable delay estimates. We appreciate the point about Figure 3C, but we felt that it was important to show the how the absolute WIS changed over time.    

 e. In both applications, nowcasts are validated using X-day-ahead rolling evaluation datasets. It is unclear why the authors did not instead evaluate predictions against consolidated values from the final version of the reporting dataset.

 > Thanks for pointing this out. We followed the recommendations from Wolffram et al to use a X-day ahead rolling evaluation dataset which were based on the analysis they performed in Section 3.7 of their manuscript, which found that using a constant lag between the time the nowast was made and the evaluation dataset more fairly evaluated nowcast performance across time in the case where very long delays can occur. We have clarified this point in the methods, stating:

 “We then quantitatively evaluated both sets of nowcasts using a rolling evaluation dataset of 40 days after each nowcast date, restricted to a maximum delay of 40 days for each reference date, consistent with the analysis performed in section 3.7 of Wolffram et al. [3] and with other nowcast evaluations [4,7,22]. This means that for each nowcast date, we used a different evaluation dataset corresponding to the data available 40 days after that nowcast date, which ensures that recent and older nowcasts are evaluated against a dataset with the same lag, reducing any bias that could be introduced from very long delays, which would systematically bias towards poorer performance in nowcasts made further in the past. “   Overall, the lack of clarity in methodology and investigation pipeline makes it impossible to determine the robustness of the results.

 > We hope that our changes based on the substantive reviewer comments have made the manuscript easier to follow.    

 2. Figure quality

 Overall figure quality is substandard. Some plots in Figure 2 appear hand-drawn, while Figures 3, 4, and 5 contain an excessive number of panels, with color schemes and legends that are difficult to map to the corresponding results. For example, in Figure 5, it is hard to assess whether any model provides qualitatively accurate nowcasts and which legend applies to panels on the right.

 >Thanks for this feedback though we don’t agree with the use of “substandard”   We agree that Figure 2 could be improved and have reorganized it to more logically guide the reader from steps 1 through 5 and replaced the components of it that were hand-drawn in powerpoint. …    We are concerned that many readers will not also read the SI and so think that the panels in 3, 4, and 5 are important to include in the main text in order to communicate the detail of our evaluation which the reviewer has highlighted is key to communicate.  We agree that the legends in Figure 5 are difficult to distinguish, we have altered the color palette of the weekdays to make them more visually distinct from the different model variations. 

 3. Information overload

 The manuscript devotes substantial space to issues of limited scientific relevance, such as the identification of implementation bugs and baseline consistency checks (e.g., Figure 3b,d). Similarly, Figures 4 and 5 and their accompanying text present a large volume of detailed results that are difficult to contextualize, especially given the lack of methodological clarity, and to summarize in its epidemiological meaning. Differences across results of model variants appear minimal, yet they are discussed at considerable length.

 > We appreciate this feedback. We hope that other changes based on earlier comments have made the results easier to digest. We agree that our results are discussed at length which we feel is a strength of the manuscript and important for evaluations of this kind in order for users to be able to trust the method. In light of this feedback, we have gone through and reviewed the results and trimmed down instances where we felt the language was overly verbose.   

 4. Claims and positioning

 In the introduction, the work positions itself as both a practical tool for public health practice and a standardized benchmark for methodological research. However, as also acknowledged in the Discussion, the paper does not succeed in achieving either objective.

 > We believe that this work does achieve the stated aims of being both a practical tool for public health practice. We worked with two different public health agencies in both the development of the tool and the analysis presented in this paper. We have revised the discussion to reflect this.   

 In summary, the work appears to be a reasonable validation exercise of an existing and previously validated model using COVID-19 data (conditional on the robustness of the methods, which would need to be clarified more clearly). The extension to norovirus data does not appear competitive relative to other existing models. The main added value of the work seems to lie in providing additional flexibility in algorithmic choices, though these are currently described unclearly, and in wrapping the method into an R package, whose usability and practical value remain to be demonstrated.

 >Thanks for this feedback. Our goal in this work was to first validate that our implementation of the baseline method used in the German Nowcast Hub (Wolffram et al), second assess the performance of the different model specifications applied to German COVID-19 data, and compare to other methods and previously used baselines applied to norovirus data. We agree that the process of improving the usability and practical feature set of the R package will be ongoing, and one which we plan to continue to develop after receiving feedback from users.

# Reviewer report: Reviewer response for version 1

**DOI:** 10.21956/wellcomeopenres.27585.r139005

**Published:** 2026-01-02

**Authors:** Felix Günther

**Affiliations:** 1 Robert Koch Institute, Berlin, Germany

Johnson et al. present a nowcasting model that allows to anticipate future changes to current real-time disease surveillance data, e.g. due to reporting delays or data revision, based on observed patterns of changes in the past. They also provide an accompanying R package, containing an implementation of the method. It allows for easy application of the method and provides flexibility in adapting it to requirements for the respective use case. The model is elegant and relatively simple, it focuses on representing the observed changes of the past in an simple way, combined with an assumption-light, empirical quantification of prediction uncertainty derived from applying the method to past time points. 

 Besides introducing the method and software, the authors illustrate application of the model based on two use cases, discuss different specifications and modelling choices and demonstrate a solid performance of the method by retrospective evaluation and comparison to alternative, more complex nowcasting methods.

 The work is motivated by the observation that nowcasting, i.e., the adjustment of disease surveillance data for reporting delays and corresponding changes in available data over time, is an important task for real-time infectious disease surveillance. In the past, several methods have been proposed for this, but they are not yet in regular use in practice. Furthermore, it is often unclear how the performance of different models differs. The new method should therefore be easy for users to apply, perform reliably, and at the same time provide a basis for comparison with the results of more complex methods.

 I enjoyed reading the article and agree with the authors that this is an important piece of work. I see the potential of the proposed method — in conjunction with the published software — to increase the applicability of nowcasting even for users who are not technical experts.

 I have two more detailed comments/questions regarding the method and it's presentation followed by some minor points and typos:

 1) The methods section in the main text of the manuscript states that training data is splitted into two non-overlapping subsets for estimating the delay distribution and uncertainty parameters (50% of the reference times, each). This seems to be appealing as it assures that the uncertainty quantification of the point predictions takes into account an "out of sample" performance/variability when comparing the nowcast point predictions to actual data. I, however, do not understand completely how this is done (I think it's not explicitly mentioned in the supplement again), and I am also wondering, whether it is really necessary. Have you also tried estimating the nowcast uncertainty and delay distribution based on the same set of reference dates and did it reduce the overall performance compared to splitting the training data? This would reduce the amount of historical data/data snapshots required for nowcasting, which could be useful in situations involving a new outbreak or data stream without much historical data. Overall, I would appreciate some more clarification of this aspect of the method.

 2) The proposed nowcasting method is described in detail in the supplementary material and explained in an understandable way with the necessary technical depth. However, when I first read the main section on the methods, I had some difficulty understanding the approach, as I felt the conceptual ideas and methodological details of the default settings were mixed up in a somewhat confusing way (e.g., I did not directly understand the necessity for the imputation of missing cells in the reporting triangle, the difference compared to the “point nowcast matrix,” and the actual nowcasting based on the currently available cumulative case numbers per reference date). I wonder, whether this section could be made more accessible by first sketching out the general idea of the method (point nowcast + uncertainty quantification based on a comparison of past point nowcasts and final data). This could then be followed by a subsection carefully describing the default choices for each step, and then the already existing subsection on the available options for model specification. I guess, such a description could help to make the basic idea of the method more accessible, also to potential users without a background in the area of nowcasting methods.

 I also have a few minor points:

 - The term "data ingestion settings" was somewhat unclear to me, might profit from clarification

 - Results: German COVID-19: The sentence "Our method had 55% coverage at 50% prediction intervals and 96% coverage at 95% prediction intervals, indicating it had slightly wider prediction intervals than observed in the data" does not exactly makes sense to me, as no prediction intervals are observed in the data. I guess a rephrasing towards the idea that empirical coverage frequencies were higher than desired would be more appropriate.

 - Discussion: "for example they can include upwards revisions", should this mean downward revisions?

 Typos: 

 - page 4: "black lines" instead of "black line"

 - denominator in Formula 5 in the supplement (first subscript t should probably be removed)

 - Supplement: Description of KIT simple nowcast implementation issue and revised nowcasts: "beign" instead of "being"

 - Supplement: "less stably" instead of "less stable"

Is the work clearly and accurately presented and does it cite the current literature?

Yes

If applicable, is the statistical analysis and its interpretation appropriate?

Yes

Are all the source data underlying the results available to ensure full reproducibility?

Yes

Is the study design appropriate and is the work technically sound?

Yes

Are the conclusions drawn adequately supported by the results?

Yes

Are sufficient details of methods and analysis provided to allow replication by others?

Partly

Reviewer Expertise:

Infectious disease modelling

I confirm that I have read this submission and believe that I have an appropriate level of expertise to confirm that it is of an acceptable scientific standard.

# Response

**Published:** 2026-02-02

**Authors:** Kaitlyn Johnson

**Affiliations:** Infectious Disease Epidemiology, London School of Hygiene and Tropical Medicine Department of Infectious Disease Epidemiology, London, England, UK

Thank you for your helpful feedback.

 We have reorganised the main text methods and added additional explanations of how the training data is used for delay and uncertainty estimation in the supplement. Please find our responses to each point below.   

 1) The methods section in the main text of the manuscript states that training data is splitted into two non-overlapping subsets for estimating the delay distribution and uncertainty parameters (50% of the reference times, each). This seems to be appealing as it assures that the uncertainty quantification of the point predictions takes into account an "out of sample" performance/variability when comparing the nowcast point predictions to actual data. I, however, do not understand completely how this is done (I think it's not explicitly mentioned in the supplement again), and I am also wondering, whether it is really necessary. Have you also tried estimating the nowcast uncertainty and delay distribution based on the same set of reference dates and did it reduce the overall performance compared to splitting the training data? This would reduce the amount of historical data/data snapshots required for nowcasting, which could be useful in situations involving a new outbreak or data stream without much historical data. Overall, I would appreciate some more clarification of this aspect of the method.  

 > Thanks for highlighting that this part isn’t clear. The training data is not strictly speaking split into two non-overlapping subsets for delay estimation and uncertainty estimation. The total reference times used for uncertainty estimation overlaps with those used for delay estimation because the uncertainty is estimated by producing a retrospective nowcast for multiple retrospective nowcast times. At each of those times, the delay is re-estimated, using the same amount of training data specified for delay estimation.   The uncertainty estimation step now reads: “We generate retrospective point nowcasts (light shaded bars and blue lines in Figure 2, step 3) by repeating steps 1 and 2 for a number of past, retrospective nowcast times, M, using N reference times for delay estimation at each retrospective nowcast time.This requires N + M total reference times, which means that the total reference times used for uncertainty estimation overlaps with those used for delay estimation, as each retrospective nowcast requires re-estimating a delay using N reference times.      We clarify this in the supplement, revising the section on uncertainty quantification to read:

 “To estimate the uncertainty in the nowcasts, we use the nowcast errors from M past nowcasting time points, with each past nowcast produced using N reference times for delay estimation. This means that the total set of reference times used for uncertainty estimation is N + M, which partially overlap with the set of reference times N used for delay estimation.”  

 2) The proposed nowcasting method is described in detail in the supplementary material and explained in an understandable way with the necessary technical depth. However, when I first read the main section on the methods, I had some difficulty understanding the approach, as I felt the conceptual ideas and methodological details of the default settings were mixed up in a somewhat confusing way (e.g., I did not directly understand the necessity for the imputation of missing cells in the reporting triangle, the difference compared to the “point nowcast matrix,” and the actual nowcasting based on the currently available cumulative case numbers per reference date). I wonder, whether this section could be made more accessible by first sketching out the general idea of the method (point nowcast + uncertainty quantification based on a comparison of past point nowcasts and final data). This could then be followed by a subsection carefully describing the default choices for each step, and then the already existing subsection on the available options for model specification. I guess, such a description could help to make the basic idea of the method more accessible, also to potential users without a background in the area of nowcasting methods.

 > We agree with the reviewer that this was confusing. We have taken the reviewers suggestions and have reorganized the methods so that we provide an overview of the method, a step-by-step description of the method conceptually, a section with the default methodological choices, a section for available options for model specification, and finally a section for the software implementation.   

 - The term "data ingestion settings" was somewhat unclear to me, might profit from clarification

 > The term is meant to refer to the amount of input training data the model uses and for what purposes (i.e. delay and uncertainty estimation). We have removed it, instead describing directly the choices that are made regarding the training data used for delay and uncertainty estimation by default and the available specification options which allow these to be modified.   

 Results: German COVID-19: The sentence "Our method had 55% coverage at 50% prediction intervals and 96% coverage at 95% prediction intervals, indicating it had slightly wider prediction intervals than observed in the data" does not exactly makes sense to me, as no prediction intervals are observed in the data. I guess a rephrasing towards the idea that empirical coverage frequencies were higher than desired would be more appropriate.

 > Thanks for catching this, this was a mistake in our description of interval coverage. We edited the sentence to state that “Our method had 55% coverage at 50% prediction intervals and 96% coverage at 95% prediction intervals, indicating that a greater proportion of observed data points fell within the prediction intervals than would be theoretically expected in a perfectly well-calibrated model (SI Figure 6,7)  

 Discussion: "for example they can include upwards revisions", should this mean downward revisions?

 > Thanks for pointing out this mistake. We have edited this accordingly.   

 We also addressed each of the typos pointed out by the reviewer and greatly appreciate the thorough attention to detail given in this review.
